# Supplementary material for: Content-rich biological network constructed by mining PubMed abstracts
Source: BMC Bioinformatics. 2004 Oct 8;5:147. doi: 10.1186/1471-2105-5-147 (PMC528731; doi:10.1186/1471-2105-5-147)
Supplement: Additional File 2 — The original results of the above study (non-essential files are deleted to keep the file size under the limit set by BMC bioinformatics). [file 1471-2105-5-147-S2.bz2 › chilibotAdditionalFile2/dip10/246ID8962075E809/html/RECO_RECF.html]

 


 **RECO** and **RECF** 
  
Found 63 abstracts in PubMed, retrieved 10.  
 

 What does Google say? 
 PDF only 
| .edu only 

---

**Interactive relationship** (e.g. stimulation, inhibition, etc)

**Neutral relationship**- **RecF**  protein competes with  **RecO**  protein for RecR protein association and its addition destabilizes RecAOR filaments.  Ref: 11743007 EMBO J, 2001

**Non-interactive relationship** (e.g. studied together, co-existance, homology, etc.)

- The Escherichia coli  **RecF** ,  **RecO**  and RecR pro teins have previously been implicated in bacterial recombinational DNA repair at DNA gaps.  Ref: 11743007 EMBO J, 2001
- ii In a recBC sbcA background, in which RecE mediated recombination is active, recA, recJ, recQ, recE, recT,  **recF** ,  **recO** , and recR mutations led to their accumulation.  Ref: 12718760 BMC Mol Biol< MedlineTA>BMC Mol Biol, 2001
- Genetic evidence suggests that the  **RecF** ,  **RecO** , and RecR RecFOR proteins participate in a common step of DNA recombination and repair, yet the biochemical event requiring collaboration of all three proteins is unknown.  Ref: 12769856 Mol Cell, 2003
- We compared the recombination proficiencies of recB1080,  **recO** , recR, and  **recF**  single mutants and recB1080  **recO** , recB1080 recR, and recB1080  **recF**  double mutants.  Ref: 12618388 Genetics, 2003
- The defects in restart attributed to mutations in  **recF** ,  **recO**  or recR were suppressed in a recA730 lexA Def strain expressing constitutively activated RecA RecA\*.  Ref: 11929519 Mol Microbiol, 2002
- Similarly,  **recF** ,  **recO**  or recR mutations suppress the lethality of the Escherichia coli rep uvrD double mutant.  Ref: 12065426 EMBO J, 2002
- Although the MRF alpha 2 components are apparently not encoded by currently known homologous recombination genes such as recA,  **recF** ,  **recO** , and recR, they may fulfill an important function in assembling replisomes on arrested replication forks and products of homologous strand exchange.  Ref: 11459960 Proc Natl Acad Sci U S A, 2001
- dnaC suppressors of priA overcome this inviability, especially when  **RecF** ,  **RecO**  or RecR is inactivated, indicating that RdgC avoids or counters a toxic effect of these proteins.  Ref: 12554673 EMBO J, 2003
- In a wild type, lexA background, mutations in  **recF** ,  **recO**  or recR block both pathways.  Ref: 11929519 Mol Microbiol, 2002
- To elucidate the role of several key proteins involved in this process, we have analysed the time dependence of replication restart in strains carrying a combination of mutations in lexA, recA, polB pol II, umuDC pol V, priA, dnaC,  **recF** ,  **recO**  or recR.  Ref: 11929519 Mol Microbiol, 2002
- previously isolated mutations in B.subtilis  **recF** , recL,  **recO**  and recR, which belong to the same complementation group, all suppress the lethality of a pcrA mutation.  Ref: 12065426 EMBO J, 2002
- This function is countered by the  **RecF**   **RecO**  competition for association with the RecR protein.  Ref: 11743007 EMBO J, 2001
- Resistance of E. coli strains to cisplatin as determined by plating experiments decreased in the following order parent PQ30 strain, polB >  **recO** , recR,  **recF**  > recB.  Ref: 12531023 DNA Repair Amst , 2002
